# Intrauterine insemination: prognostic factors

**DOI:** 10.5935/1518-0557.20240017

**Published:** 2024

**Authors:** Carla Maria Franco Dias, Gabriel Borges Tavares Vitorino, Suelen Maria Parizotto Furlan, Rosana Maria dos Reis, Ana Carolina Japur de Sá Rosa e Silva, Maria Célia Mendes, Rui Alberto Ferriani, Paula Andrea Navarro

**Affiliations:** 1 Sector of Human Reproduction, Department of Gynecology and Obstetrics - Ribeirão Preto Medical School, University of São Paulo, Brazil; 2 National Institute of Hormones and Women’s Health, CNPq, Brazil

**Keywords:** infertility, intrauterine insemination, prognostic factors, clinical pregnancy rate, live birth rate

## Abstract

**Objective:**

To evaluate the impact of possible maternal and paternal prognostic factors and ovarian stimulation protocols on clinical pregnancy and live birth rates in intrauterine insemination (IUI) cycles.

**Methods:**

Retrospective observational study of 341 IUI cycles performed from January 2016 to November 2020 at the Assisted Reproduction Service of the Clinics Hospital of the Ribeirão Preto Medical School, University of São Paulo. Clinical pregnancy and live birth rates and their potential prognostic factors were evaluated. Wilcoxon’s non-parametric test was used to compare quantitative variables, and the chi-square test to compare qualitative variables, adopting a significance level of *p*<0.05. A logistic regression model was performed to verify which exploratory variables are predictive factors for pregnancy outcome.

**Results:**

The ovulation induction protocol using gonadotropins plus letrozole (*p*=0.0097; OR 4.3286, CI 1.3040 - 14.3684) and post-capacitation progressive sperm ≥ 5million/mL (*p*=0.0253) showed a statistically significant correlation with the live birth rate. Female and male age, etiology of infertility, obesity, multifollicular growth, endometrial thickness ≥ 7 mm, and time between human chorionic gonadotropin administration and IUI performance were not associated with the primary outcomes. In the group of patients with ideal characteristics (women aged< 40 years, BMI < 30 kg/m^2^, antral follicle count ≥ 5, partner aged< 45 years, and post-capacitation semen with progressive spermatozoa ≥ 5 million/mL), the rate of clinical pregnancy was 14.8%, while that of live birth, 9.9%.

**Conclusions:**

In this study, the ovulation induction protocol with gonadotropins plus letrozole and post-capacitation progressive sperm ≥ 5 million/mL were the only variables that significantly correlated with intrauterine insemination success.

## INTRODUCTION

It is estimated that 8 to 12% of couples worldwide suffer from infertility (Agarwal *et al*., 2021), which makes this condition a significant public health concern. Its diagnosis is surrounded by fear, anxiety, and pain. However, the growing popularization of assisted reproduction techniques has enabled more and more couples to have access to infertility treatments (Ashrafi *et al.,* 2013; Fauque *et al*., 2014). Among the various options available, intrauterine insemination (IUI) has been widely adopted as the first treatment approach, depending on the underlying cause, as it is less invasive and costly compared to other techniques, such as in vitro fertilization (IVF) (Dilbaz *et al*., 2011; Cohlen *et al*., 2018).

IUI is indicated in cases of unexplained infertility, male subfertility, unilateral tubal obstruction, cervical dysfunction, anovulation, and minimal and mild endometriosis (Van Voorhis *et al*., 2001). Despite well-established indications, the success rate of IUI is relatively low when compared to other assisted reproduction techniques. Data from the Assisted Reproduction Service of the Clinics Hospital of the Ribeirão Preto Medical School, University of São Paulo (HC-FMRP/USP) showed a pregnancy rate per cycle of 12.74%, from 2011 to 2015, in couples with ideal conditions for performing IUI (Sicchieri *et al*., 2018), while the pregnancy rate per IVF cycle was 34.5% (De Geyter *et al.,* 2018).

In addition to being indicated for distinct causes of infertility, there are different protocols for performing IUI. The procedure can be carried out during a natural ovulatory cycle or after ovulation induction with oral medications and/or injectable gonadotropins (Practice Committee of the American Society for Reproductive Medicine, 2020). Ovarian stimulation aims to ensure a greater number of ovulated oocytes per cycle (Practice Committee of the American Society for Reproductive Medicine, 2020), increasing the likelihood of pregnancy. Another variation in the protocols is related to the time for performing the IUI, which can be conducted 24 hours after the spontaneous appearance of the LH surge or after 24 to 40 hours of the application of exogenous human chorionic gonadotropin (hCG) as a trigger for ovulation. There are also protocol variations regarding different seminal sample preparation techniques, different inseminated volumes, different catheters, among others. In view of the range of variations, the scientific literature still does not have solid conclusions that define the best method and the real influence of each one on the IUI outcome (Practice Committee of the American Society for Reproductive Medicine, 2020).

In order to optimize the chances of IUI success and offer couples who seek this treatment actual probabilities of positive results, it is crucial to identify the factors that influence their outcomes (Starosta *et al*., 2020). Indeed, many prognostic factors are potentially associated with IUI outcomes, including paternal age and BMI, the total number of sperm in the seminal sample, sperm morphology, sperm count with progressive motility, inseminated sperm count, maternal age and BMI, etiology of female infertility, endometrial thickness, number of mature follicles per cycle, and duration of infertility, in addition to protocol variations of the method (Dinelli *et al*., 2014; Fauque *et al*., 2014; Ghaffari *et al*., 2015; Starosta *et al.,* 2020).

In October 2016, a new low-complexity infertility outpatient clinic was implemented at the Assisted Reproduction Service of the Clinics Hospital of the Ribeirão Preto Medical School, University of São Paulo (HC-FMRP/USP), aiming at reducing the long wait list of patients seeking public assistance for the treatment of marital infertility. As a result, some changes were made in the criteria for indicating IUI, which became the first line of infertility treatment for couples eligible for this therapeutic approach. In order to favor the indication of low-complexity methods, the aim of the present study was to analyze all the IUI cycles performed at the service from January 2016 to December 2020 and identify the potential prognostic factors associated with this procedure.

## MATERIALS AND METHODS

### Study design

This was a retrospective observational study of 341 IUI cycles performed from January 2016 to November 2020 at the Human Reproduction Center of HC-FMRP/USP. All collected data were obtained through the analysis of the service’s medical records. The study was conducted in accordance with the guidelines defined by the Research Ethics Committee (CEP) of HC-FMRP/USP and the principles of the Declaration of Helsinki. The need to provide free and informed consent was waived due to the study’s retrospective nature, thus ensuring the anonymity of the research participants.

All couples included in the study underwent a basic workup to determine the cause of infertility. The following variables were evaluated: the type of infertility, primary or secondary; the age of the women and their partners; female body mass index (BMI); menstrual regularity and dosage of the hormones FSH (3^rd^ to 5^th^ day of the menstrual cycle), prolactin, and TSH to detect ovulatory and thyroid dysfunctions; seminal quality on spermogram; ovarian reserve, assessed by antral follicle count (AFC) on transvaginal ultrasound; uterine cavity and tubal permeability, through pelvic ultrasound and hysterosalpingography/hysterosonography and/or hysteroscopy and/or videolaparoscopy. In addition, all couples underwent serological tests to detect syphilis, hepatitis B and C, HTLV 1 and 2, and HIV 1 and 2.

Once the underlying cause of infertility and possible prognostic factors were established, the couples received counseling regarding the costs and benefits of assisted reproduction treatments. Those with at least one patent uterine tube and spermogram with a total progressive spermatozoon (TPS) count ≥ 3 million and/or concentration of progressive spermatozoa in the recovered sperm post-capacitation ≥ 5 million/mL were deemed eligible for IUI.

### Ovulation induction

Ovulation induction, oocyte maturation, and luteal phase support were performed according to the standard protocols used at the Assisted Reproduction Service of HC-FMRP/USP.

The induction of ovulation was carried out using five distinct protocols: isolated clomiphene citrate, isolated letrozole, isolated gonadotropins, clomiphene associated with gonadotropins, and letrozole associated with gonadotropins.

Isolated clomiphene citrate was administered at a dose of 50 to 100 mg/day for five days starting on the 2^nd^ or 3^rd^ day of the menstrual cycle or after five days of interruption of hormonal contraception (combined oral contraceptive, progestogen-only pill, or estradiol valerate).

Meanwhile, isolated letrozole was provided at a dose of 5 mg/day for five days as of the 2^nd^ or 3^rd^ day of the menstrual cycle or after five days of hormonal contraception interruption.

In turn, the gonadotropins, menotropin (Menopur^®^) or recombinant FSH (Gonal^®^ or Puregon^®^), were administered at a dose of 50 to 75 IU, on consecutive or alternate days, as of the 2^nd^ or 3^rd^ day of the menstrual cycle or after five days of hormonal contraception interruption.

The association of the oral inducer and the gonadotropins used clomiphene citrate at a dose of 50 to 100 mg/day or letrozole 5 mg/day for five days starting on the 2^nd^ or 3^rd^ day of the menstrual cycle or after five days of hormonal contraception interruption combined with the selected gonadotropin at a dose of 75 IU every other day on the 2^nd^ and 4^th^ day of induction, daily, or every other day as of the 6^th^ day of induction.

Follicular growth was monitored by endovaginal ultrasonography, starting around the 8^th^ day of ovulation induction. When a larger follicle with a mean diameter of 17 to 18 mm was detected, triggering was indicated for final follicular and oocyte maturation with urinary human chorionic gonadotropin (hCG) (Choriomon^®^, 5000 IU) or recombinant hCG (Ovidrel^®^, 250 mcg), with IUI being performed 24 to 40 hours later. The luteal phase was supplemented with either micronized progesterone (Utrogestan^®^, 200 mg/day) or dydrogesterone (Duphaston^®^, 20 mg/day).

In cases of ovulation induction for in vitro fertilization (IVF) using gonadotropins (150 to 300 IU/day), IUI was performed only when there was recruitment and growth of only one or two follicles, in cases where the patient had at least one patent tube.

At this stage, the following variables were evaluated: AFC at the beginning of treatment; the ovulation induction protocol used; the number of follicles ≥ 15 mm on the day of hCG administration; the number of hours between hCG administration and IUI performance; the duration of ovulation induction; the dose of gonadotropin used, and endometrial thickness on the day of IUI.

### Semen preparation and intrauterine insemination

The semen was prepared mainly by density gradient (90.9%/n=310 cycles). Sperm Washing was performed in 25 cycles (7.3%) and Swim-up in only one (0.3%). Cases lacking information on the mode of seminal preparation were excluded in the comparative analysis between methods and primary outcomes.

The density gradient centrifugation technique was carried out to determine the progressive motility of the sample. A volume of 1.0 mL of each colloidal suspension was added in samples with ≥ 30% progressive sperm and 0.5 mL in samples with < 30%. In the first centrifugation step, 1.0 to 0.5 mL of 90% colloidal suspension was added, followed soon after by another 1.0 to 0.5 mL of 45% colloidal suspension. Next, a maximum volume of 3.0 mL of liquefied semen was deposited over the solution. The final sample was centrifuged for 30 minutes at 1,000 rpm, with the supernatant being discarded and the pellet homogenized in 2.0 mL of MHM-C medium + 10% SSS. A second centrifugation was performed to eliminate residual particles from the colloidal gradient. Subsequently, the supernatant was discarded, and the resulting pellet was diluted in 0.5 mL of MHM-C + 10% SSS.

After the seminal preparation, the new concentration and motility of the samples were determined, obtaining the concentration of post-capacitation progressive spermatozoa. Insemination was conducted using a LABORATOIRE CCD catheter (Paris-France), with the aid of a transabdominal pelvic ultrasound.

### Statistical analyses

Initially, exploratory data analysis was carried out using measures of central position and dispersion. Qualitative variables were summarized considering absolute and relative frequencies. The chi-square test was applied to verify which independent qualitative variables were associated with the outcomes of pregnancy and live birth. In order to assess which of the quantitative variables differed statistically between the groups pregnancy (yes or no) and live birth (yes or no), the Wilcoxon test for independent samples was applied, a non-parametric test used when the assumptions of the Student’s t-test were not met. Statistical analyses were conducted using the SAS 9.4 program, and the significance level was set at *p*<0.05. A logistic regression model was performed to verify which exploratory variables were predictive factors for pregnancy outcome.

## RESULTS

Three hundred and forty-one intrauterine insemination cycles were performed from January 2016 to November 2020. The clinical pregnancy rate per cycle was 8.5% (n=29), and the live birth rate was 5.3% (n=18). There were two cases of stillbirths and two multiple pregnancies. The mean female age was 35±4.12 years.

According to our findings, the leading etiologies related to infertility were male factors (34%/n=116), unexplained infertility (26.1%/n=89), polycystic ovary syndrome (PCOS) (17.3%/n=59), low ovarian reserve (13.5%/n=46), unilateral tubal obstruction (9.1%/n=31), endometriosis grade 1 or 2 (7.3%/n=25), endometriosis grade 3 or 4 (3.8%/n=13), independent production (0.6%/n=2), and other factors (18.5%/n=63). No specific cause of infertility showed a statistically significant correlation with the primary outcomes (Table 1).

**Table 1:** Infertility factors in IUI cycles and primary outcomes.

Infertility factor	Clinical pregnancy		*p*-value	OR^*^	CI^*^	Live birth		*p*-value	OR^*^	CI^*^
	No	Yes				No	Yes			
**Unexplained infertility** No Yes	233 79	19 (7.5%) 10 (11.2%)	0.2825	1.5523	0.6925 - 3.4794	242 81	10 (4%) 8 (9%)	0.0686	2.3901	0.9123 - 6.2619
**Male factor** No Yes	207 105	18 (8%) 11 (9.5%)	0.6419	1.2048	0.5490 - 2.6439	213 110	12 (5.3%) 6 (5.2%)	0.9498	0.9682	0.3538 - 2.6493
**Endometriosis 1 and 2** No Yes	289 23	27 (8.5%) 2 (8%)	0.9252	0.9308	0.2081 - 4.1621	299 24	17 (5.4%) 1 (4%)	0.7665	0.7328	0.0935 - 5.7453
**Endometriosis 3 and 4** No Yes	299 13	29 (8.8%) 0 (0%)	0.2624	_	_	310 13	18 (5.5%) 0 (0%)	0.3855	_	_
PCOS^*^ No Yes	259 53	23 (8.2%) 6 (10.2%)	0.6141	1.2748	0.4951 - 3.2826	267 56	15 (5.3%) 3 (5.1%)	0.9416	0.9536	0.2671 - 3.4044
**Unilateral tubal obstruction** No Yes	282 30	28 (9%) 1 (3.2%)	0.2691	0.3357	0.0441 - 2.5558	293 30	17 (5.5%) 1 (3.2%)	0.5919	0.5745	0.0738 - 4.4694
**Low reserve** No Yes	268 44	27 (9.2%) 2 (4.4%)	0.2772	0.4512	0.1036 - 1.9648	278 45	17 (5.8%) 1 (2.2%)	0.3113	0.3634	0.0472 - 2.7982
**Independent production** No Yes	310 2	29 (8.6%) 0 (0%)	0.6654	_	_	321 2	18 (5.3%) 0 (0%)	0.7378	_	_
**Other factor** No Yes	250 62	28 (10.1%) 1 (1.6%)	0.0293	0.1440	0.0192 - 1.0790	261 62	17 (6.1%) 1 (1.6%)	0.1467	0.2476	0.0323 - 1.8963

* CI: confidence interval; OR: odds ratio; PCOS: Polycystic Ovary Syndrome.

At the beginning of the IUI cycle and during ovarian stimulation, the following potential prognostic factors were evaluated: female age < 40 years (88.3%/n=301), male age < 45 years (95.8%/n=322), primary infertility (71.9%/n=245), body mass index (BMI) < 30 kg/m^2^ (75.4%/n=221), absence of ultrasonographic findings of deep endometriosis (96.8%/n=329), good ovarian reserve (86%/n=288), multifollicular growth (32.8%/n=111), endometrial thickness on the day of IUI of ≥ 7 mm (84%/n=279), and time between hCG application and IUI of ≤ 24 hours (69.3%/n=205). There was no statistically significant correlation between the above factors and clinical pregnancy and live birth rates (Table 2). Regarding the ovarian stimulation protocols, a statistically significant difference was observed in relation to the live birth rate, with better results in the group that used the letrozole protocol associated with gonadotropins (*p*=0.0097; OR 4.3286, CI 1.3040 - 14.3684) (Table 2).

**Table 2:** Prognostic factors for IUI related to primary outcomes.

Prognostic factor	Clinical pregnancy		p-value	OR^*^	CI^*^	Live birth		p-value	OR^*^	CI^*^
	No	Yes				No	Yes			
**Female age** < 40 years ≥ 40 years	273 39	28 (9.3%) 1 (2.5%)	0.1473	0.25	0.0331 - 1.8896	283 40	18 (6%) 0 (0%)	0.1120	_	_
**Male age** < 45 years ≥ 45 years	293 14	29 (9%) 0 (0%)	0.2401	_	_	304 14	18 (5.6%) 0 (0%)	0.3632	_	_
**Type of infertility** Primary Secondary	226 86	19 (7.8%) 10 (10.4%)	0.4281	1.3831	0.6184 - 3.0935	231 92	14 (5.7%) 4 (4.2%)	0.5654	0.7174	0.2301 - 2.2368
**Obesity** No Yes	201 68	20 (9%) 4 (5.6%)	0.3477	0.5912	0.1952 - 1.7906	209 68	12 (5.4%) 4 (5.6%)	0.9675	1.0245	0.3198 - 3.2819
**Endometrioma** No Yes	298 11	29 (8.9%) 0 (0%)	0.3016	_	_	309 11	18 (5.5%) 0 (0%)	0.4239	_	_
AFC^*^ < 5 ≥ 5	45 261	2 (4.3%) 27 (9.4%)	0.2471	2.3276	0.5348 - 10.1308	46 271	1 (2.1%) 17 (5.9%)	0.2872	2.8856	0.3749 - 22.2112
**Follicles ≥ 15 mm** 0 1 ≥ 2	1 206 102	0 (0%) 20 (8.9%) 9 (8.1%)	0.9295	_	_	1 213 106	0 (0%) 13 (5.8%) 5 (4.5%)	0.3894	_	_
**Hours between hCG and IUI** ≤ 24 hours > 24 hours	188 85	17 (8.3%) 6 (6.6%)	0.6143	0.7806	0.2973 - 2.0496	195 87	10 (4.9%) 4 (4.4%)	0.8568	0.8966	0.2736 - 2.9375
**IUI endometrial thickness** < 7 mm^*^ ≥ 7 mm^*^	49 257	4 (7.5%) 22 (7.9%)	0.9331	1.0486	0.3462 - 3.1765	50 266	3 (5.7%) 13 (4.7%)	0.7551	0.8145	0.2239 - 2.9627
COS^*^ protocol Clomiphene Letrozole Gonadotropin Clomiphene and gonadotropin Letrozole and gonadotropin	52 28 170 34 20	5 (8.8%) 1 (3.45%) 13 (7.1%) 5 (12.8%) 4 (16.7%)	0.9368 0.3075 0.3184 0.3045 0.1371	1.0417 0.3622 0.6787 1.7034 2.3360	0.3800 - 2.8556 0.0475 - 2.7639 0.3158 - 1.4585 0.6098 - 4.7581 0.7407 - 7.3671	55 29 175 35 20	2 (3.5%) 0 (0%) 8 (4.4%) 4 (10.3%) 4 (16.7%)	0.5126 0.1838 0.4202 0.1396 0.0097	0.6091 _ 0.6766 2.3510 4.3286	0.1361 - 2.7252 - 0.2603 - 1.7584 0.7331 - 7.5392 1.3040 - 14.3684

* AFC: antral follicle count; CI: confidence interval; COS: controlled ovarian stimulation; OR: odds ratio; mm: millimeters.

Finally, the quantitative and qualitative characteristics of the spermogram prior to IUI and sperm capacitation on the day of IUI were evaluated. Among the assessed seminal parameters, only concentrations of progressive spermatozoa in the semen recovered after capacitation ≥ 5 million/mL showed a statistically significant correlation with the live birth rate (*p*=0.0253). As there were no live births from patients with motile sperm post-capacitation < 5 million/mL, it was not possible to estimate the *odds ratio* related to this variable (Table 3). Other factors, such as total sperm count, concentration, vitality, motility, morphology, and TPS, showed no statistical correlation with clinical pregnancy or live births (Table 4).

**Table 3:** Seminal characteristics of the spermogram and post-sperm capacitation and primary outcomes.

Spermogram and post-sperm capacitation	Clinical pregnancy		*p-*value	OR^*^	IC^*^	Live birth		*p*-value	OR^*^	CI ^*^
	No	Yes				No	Yes			
TPS^*^ IUI < 3 million ≥ 3 million	25 279	0 (0%) 29 (9.4%)	0.1083	**_**	**_**	25 290	0 (0%) 18 (5.8%)	0.2139	**_**	**_**
**Progressive Sperm Recovered for IUI** < 5 million/mL ≥ 5 million/mL	62 222	3 (4.6%) 26 (10.5%)	0.1464	2.4204	0.7090 - 8.2626	65	0 (0%)	0.0253	**_**	**_**
						230	18 (7.3%)			

* CI: confidence interval; mL: milliliters; OR: odds ratio; SD: standard deviation; sptz: sperm; TPS: total progressive motile sperm.

**Table 4:** Quantitative seminal characteristics of the spermogram and primary outcomes.

Spermogram and post-sperm capacitation	Clinical pregnancy		*p*-value	Live birth		*p*-value
	No	Yes		No	Yes	
Total sptz^*^ pre-IUI (mean ± SD^*^) x 10 million	171.1±160.15	193.5±158.4	0.3512	173.8±162.9	99.96±147.1	0.5839
Sptz concentration pre-IUI (mean ± SD) x 10 million/mL^*^	62.25±47.8	64.25±56.95	0.8045	62.5±48.2	61.76±55.17	0.7821
**Vitality pre-IUI (%) (mean ± SD)**	77.95±10.6	76.8±7.1	0.1576	77.9±10.4	76.2±7.9	0.1997
**Motility pre-IUI (%) (mean ± SD)**	33±15.4	37.9±16.4	0.2004	33.3±15.6	34.3±13.97	0.7712
**Kruger morphology pre-IUI (%) (mean ± SD)**	3.06±5.03	2.5±2.07	0.5366	3.04±4.96	2.5±1.96	0.7563
TPS^*^ pre-IUI (mean ± SD) x 10 million	67.2±89.4	69.8±77.3	0.5225	68.4±90.45	51.97±36.7	0.8521

* mL: milliliters; SD: standard deviation; sptz: sperm; TPS: total progressive motile sperm.

Women < 40 years old, with BMI < 30 kg/m^2^, AFC ≥ 5, and partners aged< 45 years and whose semen had post-capacitation progressive spermatozoa concentrations ≥ 5 million/mL (n=121) were considered ideal candidates for performing IUI. In this subgroup, the clinical pregnancy rate was 14.8% (n=18), and the live birth rate was 9.9% (n=12).

## DISCUSSION

Highly complex assisted reproduction techniques, including IVF and ICSI, have evolved significantly in recent years. However, low-complexity techniques, such as intrauterine insemination (IUI), maintain low and virtually unchanged success rates (Practice Committee of the American Society for Reproductive Medicine, 2020). The Latin American Network of Assisted Reproduction (REDLARA) reported a clinical pregnancy rate per IUI cycle in 2013 of 14.91% (Zegers-Hochschild *et al*., 2016), while the European Society for Human Reproduction and Embryology (ESHRE) estimated the live birth rate in 2014 to be 8.5% (De Geyter *et al*., 2018). In our study, the sample presented a clinical pregnancy rate of 8.5% and a live birth rate of 5.3%, similar to other observational studies (Vargas-Tominaga *et al*., 2020; Sicchieri *et al.,* 2018). Despite these low success rates, IUI is still considered an initial strategy among infertile couples due to its lower complexity and financial costs (Cohlen *et al*., 2018). It is noteworthy to establish prognostic factors that reinforce its indication; however, the available literature on the subject remains contentious (Guan *et al*., 2021).

Although several factors potentially related to the prognosis of IUI were analyzed in the present study, only the ovarian stimulation protocol with gonadotropins associated with letrozole was considered a predictive factor for the increase in the live birth rate after IUI. Similar results favorable to this protocol have been reported in previous research (Guan *et al*., 2021; Vargas-Tominaga *et al*., 2020). Recent studies have evidenced the superiority of ovarian stimulation with injectable gonadotropins over oral inducers (Wessel *et al*., 2022; Zolton *et al.,* 2020; Danhof *et al*., 2018; Erdem *et al*., 2015; Diamond *et al*., 2015). However, the latter are still considered the first choice in IUI on account of their lower cost, lower rate of cycle cancellation due to multifollicular growth, and lower risk of multiple pregnancies (Starosta *et al*., 2020; Zolton *et al*., 2020; Hansen, 2020; Practice Committee of the American Society for Reproductive Medicine, 2020). Studies conducted with low doses of gonadotropins (<150 IU/day) and strict cancellation criteria have shown gestational outcomes similar to oral inducers (Danhof *et al.,* 2018; Huang *et al*., 2018). The association between low-dose gonadotropins and oral inducers has also been described as an interesting strategy, as it optimizes follicular and endometrial growth while significantly reducing costs, which are often limiting (Hembram *et al*., 2017; Healey *et al*., 2003).

Another important parameter in determining IUI success is seminal quality, given that fertilization occurs *in vivo* in this technique (Agarwal *et al*., 2021; Ombelet *et al*., 2014; Van Voorhis *et al*., 2001). The concept of mild, moderate, and severe male factors remains controversial in the literature (Cohlen *et al*., 2018). Some studies indicate a cut-off value for the indication of IUI of TPS ≥ 3 million (Bensdorp *et al*., 2007), while others ≥ 10 million (Akanji Tijani & Bhattacharya, 2010). The literature review conducted by Starosta *et al*. (2020) revealed that there is still a benefit in performing IUI with values of progressive spermatozoa after seminal washing ≥ 1 million/mL. In the present study, only concentrations of retrieved motile sperm post-capacitation ≥ 5 million/mL had a statistically significant impact on the live birth rate, although it was not possible to calculate the *odds ratio*. Despite lacking statistical significance, the TPS was clinically relevant and was directly related to the concentration of retrieved spermatozoa post-capacitation, with clinical pregnancy and live birth rates in the group with ≥ 3 million spermatozoa of 9.4% and 5.8%, respectively, and 0% of clinical pregnancy and live births in the < 3 million group, corroborating previous studies (Vargas-Tominaga *et al*., 2020; Akanji Tijani & Bhattacharya, 2010). The impact of seminal preparation techniques remains uncertain (Agarwal *et al*., 2021; Starosta *et al.,* 2020; Cohlen *et al*., 2018; Boomsma *et al*., 2019).

Another relevant data in our sample, but without statistical significance, was male age. Clinical pregnancy and live birth rates in men aged < 45 years were 9% and 5.6%, respectively. In contrast, no pregnancy was observed in partners aged ≥ 45 years. The negative impact of paternal aging, especially in individuals above the age of 40, has been well documented in the literature, with a higher prevalence of miscarriage and infertility, among other effects. However, there are still inconsistencies in data in several studies (Starosta *et al*., 2020). Regarding the female age, we did not find statistically significant association with pregnancy outcomes. However, it was possible to note a clinically relevant difference between women aged <40 years and ≥ 40 years, with clinical pregnancy rates of 9.3% *vs*. 2.5%, respectively, and live births rates of 6% *vs*. 0%, respectively. The deleterious effect of aging on female fertility is widely known (Practice Committee of the American Society for Reproductive Medicine & Society for Reproductive Endocrinology and Infertility, 2017). REDLARA described a clinical pregnancy rate in IUI cycles of 18.4% in women ˂ 35 years, 13.4% in women aged between 35 and 39 years, 7.1% between 40 and 42 years, and 3.5% after 42 years (Zegers-Hochschild *et al*., 2016). Other studies, however, did not show the same correlation between female age and IUI success (Ashrafi *et al*., 2013; Erdem *et al*., 2008; Tomlinson *et al*., 1996).

Ashrafi *et al*. (2013) described a negative impact related to longer time of infertility and unifollicular growth (22.5% multifollicular *vs*. 6.5% unifollicular), similar to the findings reported in other studies (Vargas-Tominaga *et al*., 2020; Cohlen *et al*., 2018). In the present sample, there was no difference between gestational success and the growth of 1 or ≥ 2 mature follicles in the ovulation induction. On the other hand, although there was no statistically significant correlation, AFC ≥ 5 at the beginning of treatment was associated with better pregnancy and live birth rates when compared to low ovarian reserve (9.4% and 5.9% *vs*. 4.3% and 2.1%, respectively).

It was also possible to observe that female obesity contributed to the reduction of clinical pregnancy rates (5.6%) and live births (5.6%), although without statistical significance. This condition is known to be associated with ovulatory disorders, worse oocyte quality, and lower endometrial receptivity, contributing to worse reproductive outcomes (Practice Committee of the American Society for Reproductive Medicine, 2021). Aydin *et al*. (2013), in their retrospective observational study of 306 couples with unexplained infertility or male subfertility, found that BMI was the most significant predictive factor of clinical pregnancy in IUI cycles. Other studies, however, have not shown the same statistically significant association (Guan *et al*., 2021). It is worth noting that the literature recommends higher doses of oral inducers and the use of gonadotropins in this population for a better ovarian response (Guan *et al.,* 2021; Starosta *et al*., 2020).

Finally, it was possible to observe worse gestational outcomes in women with endometriosis and PCOS when compared to unexplained infertility or mild male factors. However, no single cause of infertility was found to have a statistically significant correlation with the primary outcomes. Therefore, it can be inferred that the indication for IUI should be individualized, considering the etiology and the combination of prognostic factors, such as those mentioned above. For some infertile couples, it is reasonably cost-effective to perform 3 to 4 cycles of IUI, followed by high-complexity treatments if unsuccessful (Practice Committee of the American Society for Reproductive Medicine, 2020; Starosta *et al*., 2020), especially in those with ideal characteristics, as observed in the present study (women < 40 years of age, BMI < 30 kg/m^2^, AFC ≥ 5, partners aged< 45 years, and semen recovery of ≥ 5 million/mL).

Some limitations of this study include the fact that it was observational and had a small sample size. In addition, being a service that attends couples from the public health network, the use of expensive medications, such as injectable gonadotropins and even letrozole, is minimal; thus, cases of severe infertility, including severe male factors and deep endometriosis, have IUI as the only financially viable option, a fact that may have influenced the results.

## CONCLUSION

In the analyzed group, the ovulation induction protocol with gonadotropins associated with letrozole and recovered spermatozoa concentrations after sperm capacitation of ≥ 5 million/mL were the only variables that significantly correlated with the success rates of intrauterine insemination. Several other prognostic factors did not show a statistically significant correlation, despite their clinical relevance. Therefore, IUI indications should be individualized. The combination of adverse infertility factors and the couple’s financial condition can be decisive in the indication or not of IUI or IVF/ICSI as the first treatment option. Further studies with larger samples may be required in order to corroborate the obtained results.
